# Evaluating metagenomics and targeted approaches for diagnosis and surveillance of viruses

**DOI:** 10.1186/s13073-024-01380-x

**Published:** 2024-09-09

**Authors:** Sarah Buddle, Leysa Forrest, Naomi Akinsuyi, Luz Marina Martin Bernal, Tony Brooks, Cristina Venturini, Charles Miller, Julianne R. Brown, Nathaniel Storey, Laura Atkinson, Timothy Best, Sunando Roy, Sian Goldsworthy, Sergi Castellano, Peter Simmonds, Heli Harvala, Tanya Golubchik, Rachel Williams, Judith Breuer, Sofia Morfopoulou, Oscar Enrique Torres Montaguth

**Affiliations:** 1 Infection, Immunity and Inflammation Department, Great Ormond Street Institute of Child Health, University College London, London, UK; 2 Genetics and Genomic Medicine Department, Great Ormond Street Institute of Child Health, University College London, London, UK; 3 Department of Microbiology, Virology and Infection Prevention & Control, Great Ormond Street Hospital for Children NHS Foundation Trust, London, UK; 4 Nuffield Department of Medicine, University of Oxford, Oxford, UK; 5 Radcliffe Department of Medicine, University of Oxford, Oxford, UK; 6 Division of Infection and Immunity, University College London, London, UK; 7 Microbiology Services, NHS Blood and Transplant, Colindale, UK; 8 Sydney Infectious Diseases Institute, Faculty of Medicine and Health, University of Sydney, Sydney, Australia; 9 Section for Paediatrics, Department of Infectious Diseases, Faculty of Medicine, Imperial College London, London, UK

**Keywords:** Clinical metagenomics, Viral diagnostics, Pathogen detection, Epidemiological surveillance, Next-generation sequencing

## Abstract

**Background:**

Metagenomics is a powerful approach for the detection of unknown and novel pathogens. Workflows based on Illumina short-read sequencing are becoming established in diagnostic laboratories. However, high sequencing depth requirements, long turnaround times, and limited sensitivity hinder broader adoption. We investigated whether we could overcome these limitations using protocols based on untargeted sequencing with Oxford Nanopore Technologies (ONT), which offers real-time data acquisition and analysis, or a targeted panel approach, which allows the selective sequencing of known pathogens and could improve sensitivity.

**Methods:**

We evaluated detection of viruses with readily available untargeted metagenomic workflows using Illumina and ONT, and an Illumina-based enrichment approach using the Twist Bioscience Comprehensive Viral Research Panel (CVRP), which targets 3153 viruses. We tested samples consisting of a dilution series of a six-virus mock community in a human DNA/RNA background, designed to resemble clinical specimens with low microbial abundance and high host content. Protocols were designed to retain the host transcriptome, since this could help confirm the absence of infectious agents. We further compared the performance of commonly used taxonomic classifiers.

**Results:**

Capture with the Twist CVRP increased sensitivity by at least 10–100-fold over untargeted sequencing, making it suitable for the detection of low viral loads (60 genome copies per ml (gc/ml)), but additional methods may be needed in a diagnostic setting to detect untargeted organisms. While untargeted ONT had good sensitivity at high viral loads (60,000 gc/ml), at lower viral loads (600–6000 gc/ml), longer and more costly sequencing runs would be required to achieve sensitivities comparable to the untargeted Illumina protocol. Untargeted ONT provided better specificity than untargeted Illumina sequencing. However, the application of robust thresholds standardized results between taxonomic classifiers. Host gene expression analysis is optimal with untargeted Illumina sequencing but possible with both the CVRP and ONT.

**Conclusions:**

Metagenomics has the potential to become standard-of-care in diagnostics and is a powerful tool for the discovery of emerging pathogens. Untargeted Illumina and ONT metagenomics and capture with the Twist CVRP have different advantages with respect to sensitivity, specificity, turnaround time and cost, and the optimal method will depend on the clinical context.

**Supplementary Information:**

The online version contains supplementary material available at 10.1186/s13073-024-01380-x.

## Background

Metagenomics, the sequencing of all genomic material within a sample, is a demonstrably powerful approach for detection of novel or unknown pathogens. Most notably, metagenomic sequencing identified the SARS-CoV-2 virus within 4 weeks of the first reported patient being hospitalized [1]. The unselective and comprehensive approach makes metagenomics attractive as a diagnostic tool. A single test that can identify any pathogen, including those that are unexpected and novel, holds much interest for clinical and public health laboratories. Since 2008, short-read metagenomics has been trialed by many groups to identify causes of fever and central nervous system diseases, including encephalitis, particularly in undiagnosed immunocompromised patients or outbreaks of unknown aetiology [2–11]. With the recent advent of rapid methods, such as sequencing with Oxford Nanopore Technologies (ONT), metagenomic approaches have been proposed as suitable for rapid detection of unexpected pathogens and antimicrobial resistance in respiratory samples from patients with complex pneumonias receiving intensive care treatment [12–18]. As an augmentation to metagenomics, oligonucleotide panels that enrich for large numbers of pathogens, while potentially reducing the possibilities for detection of an unknown pathogen, have been reported to improve the sensitivity and speed with which known pathogen genomes are detected, making them potentially valuable for infection diagnosis and screening [19–24].

Comprehensive evaluation of these pipelines is vital for their wider uptake in clinical laboratories. A major problem for the routine use of metagenomics in the diagnosis of infection has been the dilemma of distinguishing true and contaminating infectious agents. This is particularly challenging where deep sequencing of material with normally low microbial abundance is required to exclude infection, for example in the differential diagnosis of encephalitis [11]. In such cases, absence of a pathogen is as important as its presence, allowing clinical teams to focus on immunomodulatory approaches that could be detrimental if infection is present. The plethora of bioinformatic tools available for interpretation of results and the lack of standardization poses further uncertainties for diagnostic labs and complicates comparison of metagenomic results, particularly if generated by different protocols [25, 26].

Several benchmarking studies have been performed comparing long and short read platforms [27–34] and associated bioinformatics methods [25, 26, 35–40] for bacterial and fungal detection. However, failure to detect viral infections may hinder the utility of metagenomic methods, particularly for the diagnosis of infections in the central nervous system and in patients with compromised immune systems, in whom serious viral infections are a major cause of morbidity and mortality [41–43]. Sensitive detection of viruses is also required in other situations, including for example, screening of blood and organs for transplantation [44] and reliably detecting pathogens of high consequence in returning travelers [45, 46]. A recent study compared viral detection in simulated low biomass samples (e.g. respiratory swabs and CSF) using Illumina, ONT and targeted methods across multiple centres [47]. However, high biomass samples, such as blood and tissue, present different technical challenges due to the high levels of host genetic material and may require different metagenomics protocols.

Many metagenomic methods advocate depletion of host nucleic acid to improve sensitivity, especially where microbial abundance is low [48, 49]. However, depletion significantly reduces host transcriptomic information, which can, when combined with pathogen metagenomics, improve accuracy of diagnosis and provide important insights that inform patient management [27, 50–53]. Human transcriptomic analysis can identify immune pathways upregulated in the host and can help distinguish between viral, bacterial, and non-infectious causes of disease, which is particularly important when no pathogens are detected through metagenomics [27, 50–53]. Nucleic acid depletion methods also reduce sensitivity to microbes without cell walls, increase contamination due to additional reagents and reduce sensitivity for detection of cell-free DNA and RNA [54, 55].

To provide a pragmatic assessment of utility for routine diagnostic viral metagenomics in samples expected to have low microbial abundance, including blood and tissue, we evaluated three commonly used metagenomic platforms and eight off-the-shelf bioinformatic methods. We established the sensitivity and limits of detection of all methods on a panel of known viral sequences. In addition, we demonstrated modifications that can be used to standardize the outputs of bioinformatic tools and minimize the presence of low-level contaminating microorganisms. This will better enable comparison between different platforms and bioinformatic tools and increase confidence in reporting results. Since combined host–pathogen genomic analysis is increasingly likely to contribute to optimum patient management, we also evaluated how well the methods preserve RNA sequences from the host transcriptome. Our goal is to provide guidance on the capabilities and drawbacks of each, for routine diagnostic use and public health screening.

## Methods

### Mock clinical samples

Mock samples were prepared to represent high-biomass samples (e.g. blood and tissue) with a clinically relevant spectrum of viral loads ranging from 60 to 60,000 gc/ml. This was achieved by performing serial dilutions of a commercial genetic material mix—the ATCC Virome Nucleic Acid Mix (ATCC, MSA-1008) (Table 1, Table S1) in a background of either human DNA, RNA, or a DNA + RNA mix. Mock samples were prepared by using commercially available human genomic DNA (Promega, 20050264) and Human Brain total RNA (Invitrogen, 20050264) at a final concentration of 40 ng/µl. Lambda DNA (Sigma, 20050264) and MS2 Bacteriophage RNA (Roche® Life Science Products, 20050264) were used as internal controls. All mock samples were spiked with the internal controls to an average CT value of 31 using a 10^−6^ dilution from the commercial stock as previously described [56]. Each mock sample type (DNA,RNA or DNA + RNA) and dilution point was prepared in large batches and then split into 10-µl single use aliquots assuring that all methods were tested using the same sample batch in order to reduce variability between experiments. Viral loads (copies per ml) were calculated by extrapolating the number of copies on a 10-µl aliquot considering an average purification elution volume of 40 µl and an average sample purification volume of 250 µl. Commercial DNA stock, ATCC nucleic acid virome mix, and mock samples were tested for the presence of TTV by qPCR using the TTV R-GENE kit (bioMérieux, 423414) according to the manufacturer’s instructions.

**Table 1:** Species composition of ATCC virome virus mix

Species	DNA or RNA	Average genome GC content	Genome length (nt)
Human mastadenovirus F	DNA	51.2	34392
Human herpesvirus 5 (cytomegalovirus, CMV)	DNA	57.1	229354
Human orthopneumovirus (respiratory syncytial virus, RSV)	− RNA	33.3	15228
Influenza B virus	− RNA	40.1	18527
Mammalian orthoreovirus 3	dsRNA	46.9	23416
Zika virus	+ RNA	50.3	10952

### Untargeted Illumina sequencing

Untargeted Illumina DNA and RNA metagenomic sequencing of the mock clinical samples was performed as previously described [56]. Two technical replicates per mock sample were performed. DNA samples underwent human CpG-methylated DNA depletion using the NEBNext® Microbiome DNA Enrichment Kit (New England Biolabs, E2612L) followed by library preparation using the NEBNext® Ultra™ II FS DNA Library Prep Kit for Illumina (New England Biolabs, E7805L). RNA samples underwent ribosomal RNA (rRNA) depletion followed by library preparation using KAPA RNA HyperPrep kit with RiboErase HMR (Roche, KK8561). For RNA-seq, DNA viruses present in the sample were removed during the DNaseI step performed during rRNA depletion protocol. All samples for untargeted Illumina sequencing were processed using xGen™ UDI-UMI Adapters (IDT, 10005903).

All pre-PCR steps were carried out under an MSC class II cabinet and moved to a post-PCR area following amplification. Libraries were quantified with high sensitivity dSDNA kit (Invitrogen, Q33231) on an Invitrogen Qubit 4 Fluorometer and the average peak sizes for libraries were checked using high sensitivity D1000 screentapes (Agilent, 5067–5584) on a Tapestation 4200. Samples were sequenced in equimolar pools using a NextSeq 2000 or a NovaSeq 6000 300 cycle kit (2 × 150 bp) depending on the number of samples processed. A minimum output of 5 Gb per sample was obtained (Table S2).

### ONT sequencing

ONT sequencing was performed using PCR-based protocols and Q20 + chemistry (Version 14 kits). Two technical replicates per mock sample were performed. DNA samples underwent human CpG-methylated DNA depletion using the NEBNext Microbiome DNA enrichment kit (New England Biolabs, E2612L) prior library preparation using the Rapid PCR Barcoding kit 24 V 14 (Oxford Nanopore Technologies, SQK-RPB114.24) according to the manufacturer’s instructions. RNA sequencing was performed using Rapid-Smart 9N [57]. Before library preparation, a DNase I (New England Biolabs, M0303S) treatment was performed to remove DNA virus present in the mock community. First, for annealing of the tagged random oligonucleotide, 10 µl of RNA was mixed with 1 μl of 2 μM RLB RT 9N oligo (TTTTTCGTGCGCCGCTTCAACNNNNNNNNN) and 1 μl 10 mM dNTPs. Mix was incubated for 5 min at 65°C, then cooled on ice. For cDNA synthesis and generation of double-tagged cDNA, 4 μl SuperScript IV First-strand Buffer, 1 μL 0.1 M DTT, 1 μl RNase OUT (Thermo Fisher Scientific, 10777019), 1 μl 2 μM RLB TSO (GCTAATCATTGCTTTTTCGTGCGCCGCTTCAACATrGrGrG), and 1 μL SuperScript IV (Thermo Fisher Scientific, 18090010) were mixed with the 12 μl annealed RNA. Reaction was incubated for 90 min at 42°C followed by 10 min at 70°C. Five microliters of double-tagged cDNA was used as input for the PCR step in the Rapid PCR Barcoding kit 24 V 14 (Oxford Nanopore Technologies, SQK-RPB114.24). From this step onwards, the manufacturer’s instructions were followed.

All pre-PCR steps were carried out under an MSC class II cabinet and moved to a post-PCR area following amplification. Sequencing was performed using PromethION Flow cells (R.10.4.1) on a P2 solo device connected to a GridION. Real-time basecalling was performed in MinKnow Version 23.07.5 using the high-accuracy model. Samples were sequenced until a minimum output of 5 Gb per sample was obtained (Table S2). ONT adaptive sampling was not used.

### Targeted Illumina sequencing with Twist Comprehensive Viral Research Panel

Targeted Illumina sequencing was performed on samples with a combined DNA + RNA background using the Twist Comprehensive Viral Research Panel (Twist Bioscience, 103550) following the Twist Bioscience Total Nucleic Acids Library Preparation EF Kit 2.0 for Viral Pathogen Detection and Characterization protocol. Two technical replicates per mock sample were processed other than for 60 and 600 gc/ml and the negative control, where four replicates were performed. Additional replicates were included to thoroughly test for potential cross-contamination and to assess potential sensitivity loss in low copy number samples when combined with high copy number samples in hybridization-capture reactions.

First, cDNA synthesis was performed using ProtoScript II First strand synthesis kit (New England Biolabs, E6560) followed by the NEBNext Ultra Non-Directional Second Strand Synthesis module (New England Biolabs, E6111) as recommended by TWIST Bioscience. Twenty nanograms of the double-stranded cDNA and dsDNA mix was used as input for adapter ligation, indexing and pre-capture amplification using the Twist Library preparation EF Kit 2.0 (Twist Bioscience, 104207 + 100573). All pre-PCR steps were carried out under an MSC class II cabinet until the indexing step was complete.

Following pre-capture amplification, indexed samples were pooled, for a total of 7 samples plus a negative control per hybridisation reaction, making a total of 8 samples per reaction as recommended by the manufacturers. Hybridisation was performed overnight for 16 h. Hybridisation targets were then captured with Streptavidin Binding Beads. At this step, samples were washed using the Twist Wash Buffers (Twist Bioscience, 104178) instead of the washing buffers V2 as per recommendation of the manufacturer. Post-capture amplification was performed on the enriched libraries (8 cycles). Final enriched libraries were quantified with Qubit high sensitivity kit and average peaks obtained with high sensitivity D1000 tapes. Samples were sequenced in equimolar pools using a NextSeq 2000 or a NovaSeq 6000 300 cycle kit (2 × 150 bp) depending on the number of samples processed. A minimum output of 5 Gb per sample was obtained (Table S2).

### Databases for taxonomic classification

Since database composition has been shown to have a significant impact on the results of metagenomics [58], a common set of sequences was used to build the databases where possible. For the tools where it was possible to create a custom database (Kraken2 [59], Bracken [60], Dragen Metagenomics Pipeline [61], EPI2ME labs wf-metagenomics [62], metaMix [63], MEGAN-LR [64] and Kaiju [65]), a database was created based on the bacterial (complete genomes only), viral, fungal, protozoa and human nucleotide from RefSeq (downloaded 6th June 2023). Databases were built using the default parameters, other than for MEGAN-LR, where the recommended settings for ONT data described in [39] were used. A common set of taxonomy files downloaded from NCBI (31st July 2023) were also used. Unplaced contigs were removed from the parasites and fungal nucleotide sequences prior to building the databases to reduce human contamination present in some of the reference sequences. It is not currently possible for the user to alter the databases for CZ ID [66] or One Codex [67], so the inbuilt databases were used.

### Read preprocessing and taxonomic classification

Reads were randomly subsampled from the raw output fastq files, using seqtk sample [68] for the Illumina data and a custom python script for the ONT data, to obtain 5 Gb for each sample across all the technologies.

Kraken2, Bracken, and Kaiju were run through the nf-core Taxprofiler pipeline [69], which aims to provide a reproducible best-practice workflow for metagenomics analysis. As recommended, read preprocessing involving adaptor trimming and complexity filtering with fastp [70] was performed for Illumina but not ONT sequencing [71]. Host removal was performed for both platforms by alignment to the human genome (version Ch38).

The reads obtained following preprocessing and host removal from the Taxprofiler pipeline were used as input to MEGAN-LR, run through the PB-metagenomics tools pipeline [72], with the adjustments for ONT sequencing recommended in [39].

For Illumina data processed with metaMix, a separate preprocessing pipeline was used for a more thorough removal of host reads. This involves read trimming using TrimGalore [73], followed by removal of human DNA/RNA and ribosomal RNA using alignment with both Bowtie2 [74] and BLAST [75, 76]. For the other classifiers, the time saved in classification was shorter than the time taken for the longer host removal pipeline, so a single alignment step is sufficient. For the ONT data, the output of the preprocessing and host removal from Taxprofiler was used as input. Reads were then aligned to the reference database with BLAST (nucleotide mode) and DIAMOND [77] (protein mode) before input to the metaMix R package. metaMix-fast is the first two steps of the metaMix R package, before the time-consuming MCMC step.

Raw reads were uploaded to CZ ID metagenomics workflow through the online interface. For CZ ID, the fields nr_count or nt_count were used for protein and nucleotide analyses respectively. Raw reads were also uploaded to the One Codex platform. The reads field from the reads field used for the analysis. For the Twist panel data, the Twist Comprehensive Viral Research Panel report was also run. Initially, this report failed to identify Reovirus, but this issue has since been rectified by One Codex.

Raw reads were also used as input into either Illumina’s Dragen Metagenomics Pipeline or ONT’s EPI2ME labs wf-metagenomics as appropriate. The same Kraken2 custom database as used for running Kraken2 through nf-core Taxprofiler was used, and both tools were run using the command-line interface.

### Alignment and sensitivity analysis

Reads were aligned to reference genomes downloaded from ACC using Bowtie2 [74] for the Illumina data and Minimap2 [78] for the ONT data, using the “very-sensitive” mode and the default parameters respectively. PCR duplicate reads were removed before calculating coverage and depth using samtools [79].

### Assembly

For metagenomic genome assembly, preprocessed and human filtered reads from Taxprofiler were assembled using either metaSPAdes [80] (Illumina and Twist CVRP) or metaFlye [81] (ONT), with the default parameters. The assembly of the resulting contigs (> 150nt only) was assessed using metaQUAST [82]. For de novo assembly, reads aligning to the viral community genomes were assembled using SPAdes [83] (Illumina and Twist CVRP) or Flye [84] (ONT). Assembly quality metrics were calculated using Quast [85]. Consensus sequences were generated using samtools consensus [79] from alignments to the viral community reference genomes. The assemblers used were chosen due to their good performance in a recent benchmarking study [86].

### Identification of false positive species

To standardize the results between classifiers for comparison, taxonomic ranks were identified, organisms were classified as bacteria, viruses, fungi or other eukaryotes, and all reads assigned to taxonomic levels below species were assigned to the relevant species, using custom R scripts and the taxonomizr package [87]. Where an organism was detected by both DNA and RNA sequencing, the result with the higher number of reads was retained, meaning that a species detected in both nucleic acid types would only be counted once. All analysis was performed in terms of reads rather than base pairs since not all classifiers output assignments by read, making it impossible to calculate base pair assignments for the ONT data. Read per million ratios (RPMR) and proportion of microbial reads (PMR) were calculated and used to identify positive species as described in the Supplementary Information. False positive species were defined as species that were identified by the classifiers that were not one of the six viral species in the mock community or either phage used as a positive control. False positive viral species were classified according to host using the Virus-Host DB [88].

### Host transcriptomic analysis

Genes and transcripts were quantified using Kallisto [89] with human genome GRCh38.p14 downloaded from Gencode [90]. Analysis was conducted in R using the tximport [91] and rtracklayer [92] packages. Spliced reads were identified by alignment to the human genome using STAR [93], using the presence of the CIGAR string to identify gapped alignments.

### Plots

Plots were produced in R using Tidyverse [94] packages or using Biorender.com.

## Results

### Sensitivity and limit of detection

**Fig. 1:**
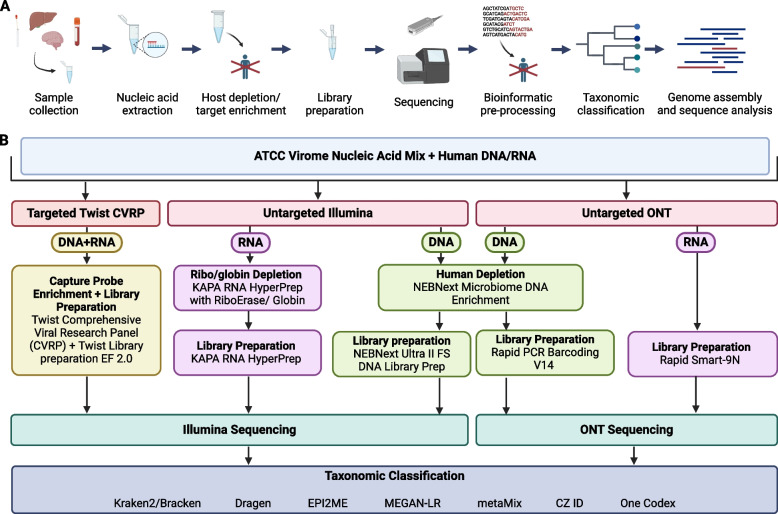
Metagenomic sequencing and experimental outline. **A** Overview of a typical clinical metagenomic processing pipeline. **B** Flow chart summarizing experimental design, which involves inputting mock and clinical samples into three metagenomic workflows: Illumina DNA and RNA seq using NEBNext and KAPA kits respectively, ONT DNA and RNA seq using the Rapid PCR barcoding kit and the Rapid Smart-9N method respectively, and finally the targeted DNA- and RNA-based Twist viral research panel, sequenced on the Illumina platform. The resulting data was analysed using different taxonomic classifiers. Produced with biorender.com

We tested simulated post-extraction clinical samples where the input viral composition is known, consisting of a mock community of genomic DNA/RNA from six viruses, two DNA (human mastadenovirus F and human betaherpesvirus 5) and four RNA (mammalian orthoreovirus, human orthopneumovirus, influenza B virus and Zika Virus), at four different concentrations in a constant human DNA and RNA background (Fig. 1). The viral loads chosen, 60–60,000 genome copies per ml (gc/ml), were designed to resemble different levels of viruses observed in clinical samples such as blood and tissue as closely as possible [95–99]. The same input was used for all metagenomics approaches: the untargeted Illumina and ONT protocols, and the capture probe enrichment with the Twist Comprehensive Viral Research Panel (CVRP) followed by Illumina sequencing (Fig. 1). At least two replicates were tested for each technology-concentration pair. We obtained 38.2–81.2 and 38.8–66.9 million reads per sample for untargeted Illumina sequencing and Illumina following the Twist CVRP respectively, corresponding to 5.7–12.2 Gb and 5.8–10.0 Gb respectively (Table S2). For the untargeted ONT sequencing, we obtained 5.1–12.3 Gb per sample. To improve comparability between methods, we randomly subsampled 5 Gb from each sample across the platforms for analysis.

The internal controls, phages lambda and MS2 for DNA and RNA respectively, were detected in all targeted and untargeted Illumina samples, other than MS2 in some of the Twist CVRP samples, which is likely due to the probes used in the panel not targeting the phage (Table S3). However, phage lambda was only found in four of the ten ONT DNA samples (Table S3). To confirm the sequencing had worked successfully, we also aligned to the human beta globin gene. Either beta globin or lambda was identified in all the samples (Table S3), and at least 5 Gb of data was obtained from all samples (Table S2), so we proceeded with analysis. During taxonomic classification, the lambda phage was not detected and was instead misclassified as *Escherichia coli* (Table S4), as several reference genomes contain an integrated lambda phage, which invalidates lambda phage as a suitable choice for DNA internal control.

**Fig. 2:**
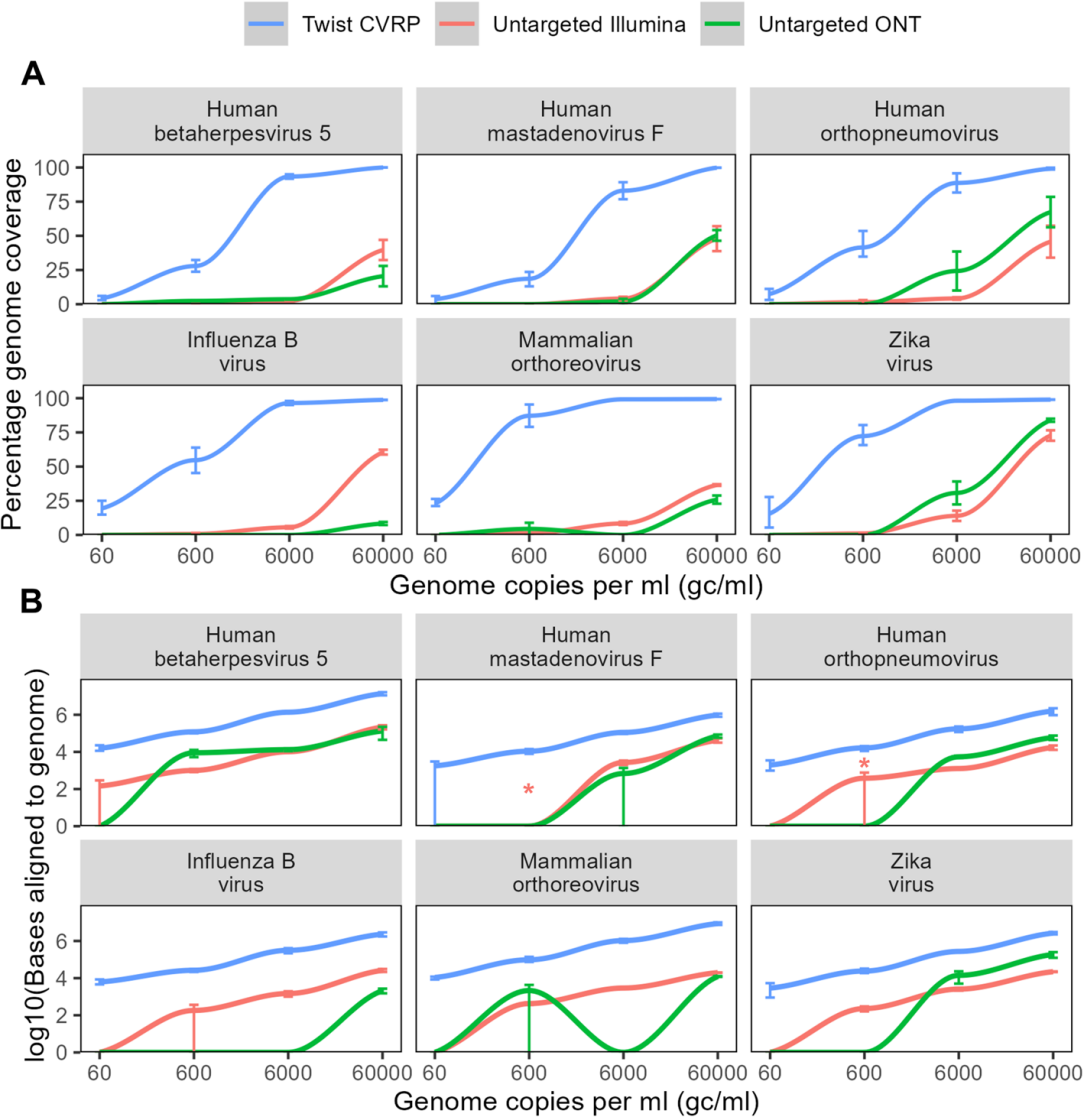
Detection of mock community viruses.  Coverage and base pairs aligned to the six expected viral species in mock samples, by untargeted Illumina and ONT sequencing and capture probe enrichment with the Twist Bioscience Comprehensive Viral Research Panel followed by Illumina sequencing. **A** Percentage genome coverage at depth 1 × of species in mock community. **B** log10(bases) aligning to reference genome. Samples where a virus was detected in the full dataset but not the subsampled dataset are indicated with a *. Genome copy numbers refer to an average across the viral species—see Table S1. Each point shows the mean of at least two technical replicates—error bars show the range. PCR duplicate reads removed

The Twist CVRP was the most sensitive method, as it was the only platform to detect all the expected viruses at 60 genome copies per ml (gc/ml), with coverage over 98.8% for all viruses at 60,000 gc/ml and ranging from 3.7 to 23.0% at 60 gc/ml (Fig. 2A). ONT was less sensitive than Illumina, detecting in at least one of the replicates all six viruses at 60,000 gc/ml, four of six viruses (human betaherpesvirus 5, human mastadenovirus F, orthopneumovirus and Zika virus) at 6000 gc/ml but only two viruses, one double-stranded (ds) DNA (human betaherpesvirus 5) and the other dsRNA virus orthoreovirus at 600 gc/ml and none at 60 gc/ml. The detection of the dsRNA virus orthoreovirus at 600 gc/ml despite not being detected at 6000 gc/ml represents only four reads in one of the replicates, with no reads detected in the other replicate, likely reflecting stochastic variation. In contrast, untargeted Illumina detected all six viruses at 60,000 and 6000 gc/ml, five at 600 gc/ml (all apart from human mastadenovirus F) and one at 60 gc/ml (human betaherpesvirus 5) (Fig. 2A). At levels close to the limits of detection, there was sometimes variation between the technical replicates in their ability to detect the viruses (Fig. 2). One additional DNA virus (human mastadenovirus F) and one additional RNA virus in one of the repeats (human orthopneumovirus) were detected by Illumina sequencing at 600 gc/ml when additional sequence data was available beyond 5 Gb (9.6 and 10.7 Gb for DNA and 11.1 Gb for RNA) (Table S2). Other than this, no additional viruses were detected in the full datasets before subsampling.

At 60,000 gc/ml, assigned bases ranged from 17,527 to 217,630 of 5 Gb for Illumina and 2110 to 134,026 of 5 Gb for ONT (Fig. 2B). Both ONT and Illumina untargeted sequencing provided incomplete coverage of the viral genomes at all concentrations tested, with percentage genome coverage at a read depth of at least 1X for the viruses in the mock community ranging from 8.4 to 83.9% at 60,000 gc/ml, 0–30.7% at 6000 gc/ml, and 0–8.9% at 600 gc/ml (Fig. 2A). Viruses with longer genomes were detected with greater read numbers; however, normalizing for genome length gave similar abundance estimates for each virus, where viral loads were high enough for consistent detection (Fig. S1). All technologies displayed levels of PCR duplication ranging from 0 to 69.7%, with the Twist CVRP showing the highest rates (Fig. S2A). The greater duplication rates with the Twist CVRP are likely explained by the additional post-capture PCR step in the Twist CVRP compared to the untargeted methods and are comparable to the rates observed in other capture panels [100]. Lower viral load samples display higher duplicate rates potentially due to the reduced amount of material available for PCR after the hybridization capture step [100]. Including PCR duplicates made no difference to the conclusions regarding sensitivity (Fig. S2B).

Where novel strains or species of viruses are detected, assembly approaches can recover viral genomes. Metagenomic de novo assembly of human-filtered reads enabled identification of contigs corresponding to the viruses in the mock community at 60,000 gc/ml, corresponding to 0–21.3%, 0–85.8% and 72.5–99.1% for untargeted Illumina, ONT and the Twist CVRP respectively (Fig. S3, Table S5). De novo assembly of reads aligning to the genome gave similar results to the metagenomic assembly, demonstrating that it is possible to assemble partial viral genomes from these samples even without knowledge of the reference sequence (Fig. S3, Table S5). Partial consensus genomes from aligned reads were generated where there was sufficient coverage (Fig. S3, Table S5).

**Fig. 3:**
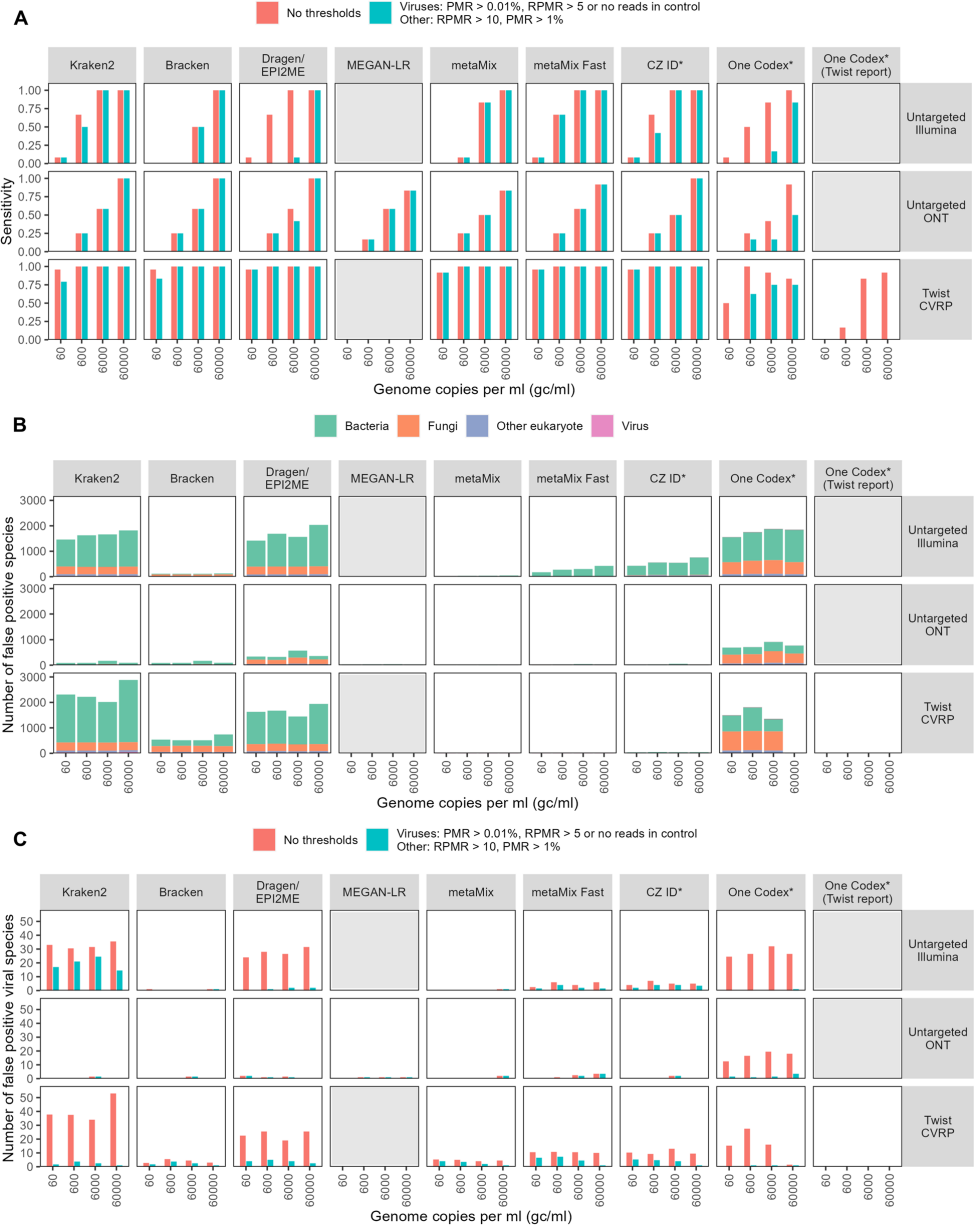
Sensitivity and number of false positive species identified by taxonomic classifiers. **A** Sensitivity to the species in the mock community before and after the application of thresholds in the legend and further defined in the Supplementary information, for seven different taxonomic classifiers, by untargeted Illumina and ONT sequencing and capture probe enrichment with the Twist Bioscience Comprehensive Viral Research Panel followed by Illumina sequencing. MEGAN-LR and the One Codex Twist report are only designed for ONT and Twist sequencing respectively so were only run for these platforms. **B**, **C** Number of false positive species, defined as a species that is classified as positive but not present in the mock community. **B** False positive species from the raw output of the taxonomic classifiers with no thresholds applied. **C** Comparison of the numbers of viral positive species identified before and after the application of thresholds. RPMR: reads per million ratio, PMR: proportion of (nonhuman classified) microbial reads—see Supplementary Information for further details. Genome copy numbers refer to an average across the viral species—see Table S1. Each bar shows the mean of at least two technical replicates

We also tested the sensitivity of a range of taxonomic classifiers. The classifiers tested and reasons for inclusion are outlined in Table 2. Where no thresholds were applied, all the classifiers had similar sensitivity, although there was some variation in ability to detect viruses at 60–6000 gc/ml for untargeted Illumina sequencing and at 60,000 gc/ml for untargeted ONT sequencing, with Kraken2, Dragen, metaMix-fast and CZ ID being the most sensitive at these viral loads (Fig. 3A). MetaMix and MEGAN-LR failed to identify influenza B virus and mammalian orthoreovirus respectively with ONT sequencing at 60,000 gc/ml; both RNA viruses for which fewer than 10 reads were detected by the aligner minimap2. Of the other classifiers, One Codex had substantially lower sensitivity for the Twist CVRP data compared to other classifiers, all of which identified almost all the viruses at all concentrations tested (Fig. 3A). This may be because the program only reports organisms that reach a set of predetermined abundance thresholds [101], which may not be reached at low viral loads, while the other classifiers do not by default use such thresholds. Where viruses were detected, the classifiers provided broadly similar estimates of reads per million, ranging, for example, from 30.3 to 73.6, 31.0 to 39.2 and 3695 to 8164 RPM, for human betaherpesvirus 5 for Illumina, ONT and the Twist CVRP respectively (nucleotide-based classifiers only) (Fig. S4).

**Table 2:** Taxonomic classifiers

Classifier	Method	Reason included	Platform​	GUI or CLI	Local or cloud​	Database size (based on identical fasta files)	Approximate time taken (hours)^a^	Reference
Kraken2 & Bracken	Kmer-based, lowest common ancestor	Very widely used	Illumina & ONT	CLI	Local	124 GB	1–3	[59, 60]
DRAGEN Metagenomics (Kraken2)	See Kraken2	Illumina’s platform	Illumina & ONT	CLI & GUI	Cloud	124 GB	1.5–3	[61]
EPI2ME Labs wf-metagenomics (Kraken2 & Bracken)	See Kraken2	ONT’s platform	ONT	CLI & GUI	Local. Cloud in development​	124 GB	0.5–2	[62]
MEGAN-LR	Lowest common ancestor	Good performance in benchmarking study [39]	Illumina & ONT	CLI required for preprocessing GUI (free), CLI (paid for short reads)	Local	327 GB (Minimap2) 88 GB (DIAMOND)	5–8	[64]
metaMix	Bayesian mixture models	Good performance in benchmarking study, used clinically [36]	Illumina & ONT	CLI	Local	148 GB (BLAST) 88 GB (DIAMOND)	5–12 +	[63]
CZ ID	Alignment and assembly	Free, cloud-based platform	Illumina & ONT	CLI & GUI	Cloud	NA – inbuilt online database​	0.5–2	[66]
One Codex	Kmer-based	Recommended platform for use with Twist CVRP	Illumina & ONT	CLI & GUI	Cloud	NA – inbuilt online database	~ 0.5–2	[67]
Kaiju	Local alignment based	Widely used protein classifier	Illumina & ONT	CLI	Local	101 GB	1–3	[65]

^a^Shows approximate range only—exact time taken depends on sample complexity, number of samples processed and computational resources, and exact timings were not available for all classifiers, e.g. One Codex. Based time taken to process single samples from our dataset, including bioinformatic preprocessing

### False positive rates

High precision and low false positive rates are as important as sensitivity in a clinical diagnostic setting, and rational approaches to identifying and reporting contaminants, particularly by non-specialist bioinformaticians, are needed. There is currently no gold-standard classifier for identification of viruses from metagenomics data and a range of programs are used in clinical services [11, 12, 102]. While many benchmarking studies of metagenomics bioinformatics pipelines have been performed, several recently developed tools [61, 66, 67, 103] designed to be run by non-bioinformaticians have so far not been evaluated in this way for detection of viruses. To evaluate their performance, we compared the number of false positive species identified by a range of commonly used taxonomic classifiers for the mock samples (Table 2). A false positive is defined as any species not present in the mock community. All the classifiers assigned similar numbers of reads to the species in the mock community, except for One Codex, which had lower sensitivity for the Twist CVRP data than the other classifiers (Fig. S4). However, when no additional thresholds were applied, there was a large variability between the classifiers in terms of the number of species identified by Illumina sequencing (Fig. 3B). Most of the false positive species were fungi or bacteria. Kraken2 Illumina’s Dragen Metagenomics Pipeline (which is based on Kraken2) and One Codex, all use kmer methodologies and identified over 1500 false positive species for the untargeted Illumina sequencing (Fig. 3B, Table S4). The discrepancy between the number of false positives identified for the Twist CVRP data by One Codex at different concentrations may be caused by greater availability of data for the classifier to distinguish between true and false positives at higher read depths [101]. By contrast, metaMix and Bracken, which both use Bayesian methods, identified only one false positive viral species at 60,000 gc/ml (Fig. 3C). However, both these classifiers were less sensitive at lower genome copy numbers than classifiers such as Kraken2 and CZ ID. In contrast to Illumina, few false positive species, especially viruses, were identified with ONT sequencing (Fig. 3B, C). Thus, for ONT the application of thresholds beyond a basic comparison to the negative control may not be required.

To reduce the number of false positive species identified for Illumina sequencing, we imposed more stringent thresholds. Completely disregarding all species with any reads in the negative control may result in a reduction in sensitivity, particularly when there is low-level cross-contamination from high viral load samples into the control. We therefore used thresholds based on reads per million ratio (RPMR), which allows a normalized comparison between assigned reads in the sample and in the negative control. However, using RPMR alone may does not deal with the very large number of organisms with less than 5 reads assigned output by some classifiers, nor does it address low-level bioinformatic contaminants that may arise when a small number of reads from one of the mock community species are misclassified as a closely related species (e.g. a small number of reads are misclassified as adenovirus C in a sample containing adenovirus F). We can overcome this by using thresholds based on calculating the proportion of total microbial reads that are assigned to a particular species. This works on the assumption that a clinically relevant organism will represent at least 1% of the total microbial reads in the case of bacteria/eukaryotes and 0.01% in the case of viruses, which is likely to be true in most clinical samples with low microbial diversity. More details of the derivation of our thresholds can be found in the Supplementary information.

We found that using a combination of reads per million ratio between sample and the corresponding negative control and proportion of microbial reads resulted in optimum sensitivity (91.7%) and specificity (77.4%), which may be useful for classifiers such as Kraken2 and One Codex which require additional thresholds (Fig. 3A, C). In contrast, ONT sequencing and classifiers such as metaMix have few false positive reads and can be used with only a comparison to the negative control. Use of protein-based classifiers, including Kaiju [65] and the protein modes of MEGAN-LR, metaMix and CZ ID, did not improve the sensitivity classification or the number of false positives identified (Fig. S5). Some false positive viruses remained after the application of these thresholds. These are unlikely to be background or laboratory contaminants, since the use of reads per million ratio will remove any species that are present at similar levels in the negative controls.

The false positive viral species that remained after the application of thresholds were mainly viruses that do not infect mammals or birds, making them unlikely to be clinically relevant (Fig. S6, Table S6). The remaining false positive viruses were mainly Anelloviridae (often Torque Teno viruses), and viruses that were related to those in the mock community, such as other herpes or adenoviruses. The Anelloviridae, which are very commonly found in human samples, were found in both negative controls using the Twist CVRP and are a result of low-level contamination of the human genetic material, which was confirmed by PCR (CT 36.7). Some TTV species were identified in the controls, but some additional TTV species were present in the samples and were therefore called as positive. This may be because some of the classifiers were unable to accurately distinguish between TTV species based on the reads that were present, or where TTV levels were low stochastic variation may have caused it to be picked up at higher levels in samples than controls. This demonstrates the limitations of using a single threshold for all viruses, particularly when viral loads are low, and highlights that careful interpretation of the results remains essential. Identification of low levels of related viruses are likely misclassifications due to high levels of similarity between the genomes of closely related viruses.

### Host transcriptomic analysis

**Fig. 4:**
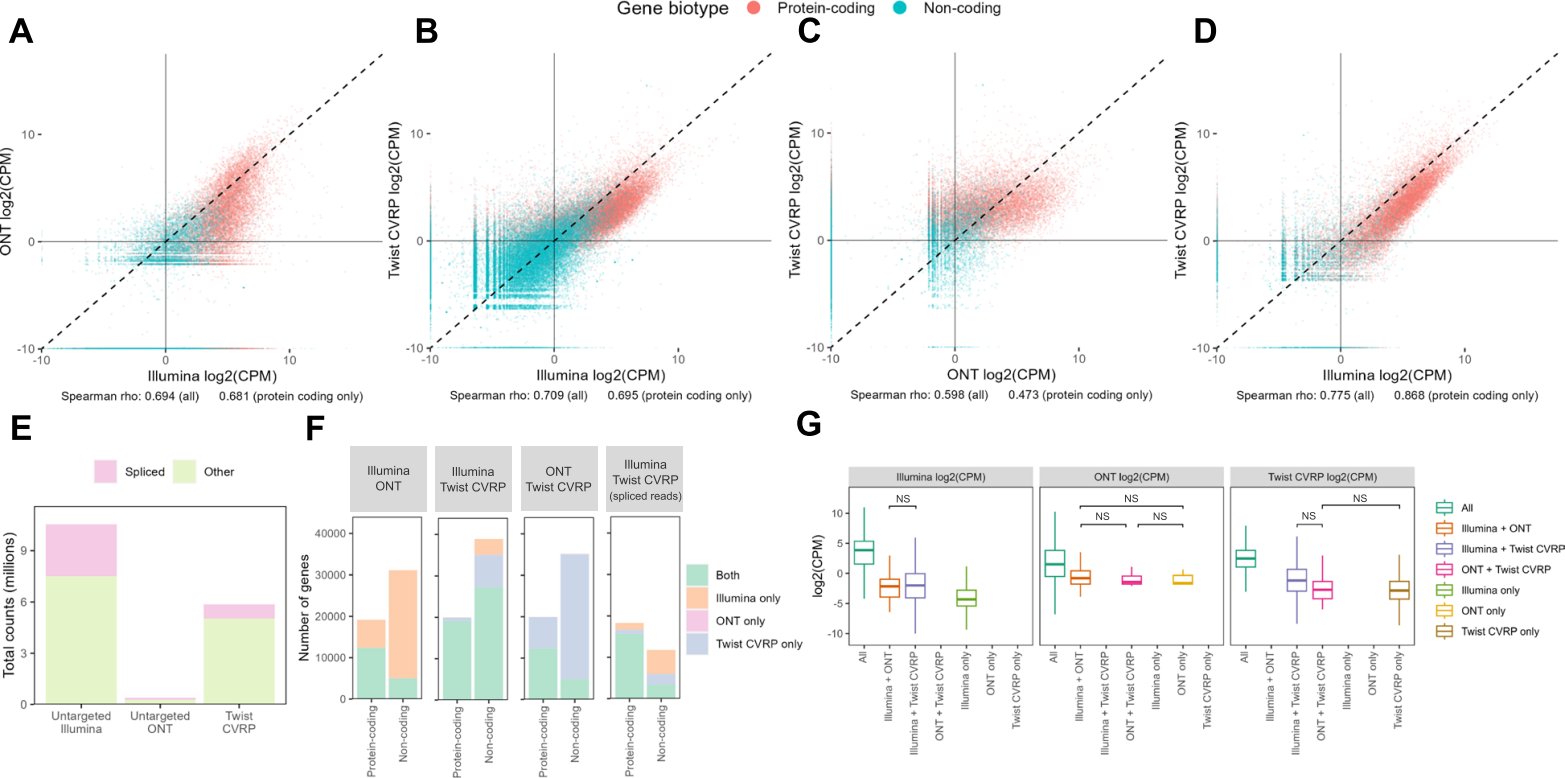
Host transcriptomic analysis. **A**–**D** Read counts per million assigned to each gene in the human genome by untargeted Illumina, untargeted ONT and targeted Illumina sequencing using the Twist Viral Research Panel. Each point represents a gene . **A**–**C** raw reads; **D** only reads that map across splice junctions. **E** Total counts for spliced and other reads. **F** Number of genes identified by each pair of technologies. **G** Counts per million of reads by platform. Each panel shows the log2(CPM) as estimated by a different technology. Outliers not shown. All comparisons are statistically significant (*p* < 0.01) with a pairwise Wilcox test other than those indicated

Several studies highlight the power of host transcriptomics methods for distinguishing bacterial, viral, and non-infectious causes of illness [27, 50–53], although none are being used diagnostically at present. When metagenomics does not identify any pathogens, such analysis could help distinguish between a non-infectious cause of disease and a lack of sensitivity of the metagenomics protocol. Since Illumina RNA sequencing has been extensively used and validated for transcriptomic studies, we compared the estimates of human gene expression provided by the ONT and Twist CVRP platforms to those from Illumina. Although the Twist CVRP only enriches for viruses, it retains the background, meaning that this analysis remains possible. The number of reads assigned to each human protein-coding gene was positively correlated between Illumina and the other two technologies (correlation coefficients, Spearman’s rho, 0.694 and 0.709 for ONT and the Twist CVRP respectively) (Fig. 4A–C). Due to the combined DNA and RNA protocol used with the Twist CVRP, there were a large number of human genes that were identified as highly expressed by the panel but not untargeted Illumina (Fig. 4B). We therefore repeated the analysis, focusing only on reads that mapped across exon-exon junctions, termed henceforth “spliced reads”, which are likely to represent mRNA, resulting in a better agreement between the Illumina and the Twist CVRP results (Fig. 4D).

While most protein-coding genes were identified by all the technologies (Fig. 4E), there was still a substantial minority that were not identified by ONT (Fig. 4E). Use of spliced reads for untargeted Illumina and Twist CVRP, only resulted in a small drop in the number of protein-coding genes identified, and a larger drop in the non-coding transcripts (Fig. 4E). However, use of the spliced reads resulted in a six-fold decrease of in the total counts for the Twist CVRP (Fig. 4E), meaning that this preliminary method to identify RNA-derived reads from a DNA-RNA mix is likely to require further refinement. However, the majority of human genes were still detected using this method **(**Fig. 4F). Genes that were identified by all technologies were significantly more highly expressed (Fig. 4G) suggesting that low-expressed genes may be less reliably identified by all technologies, particularly ONT.

### Turnaround time and cost

**Fig. 5:**
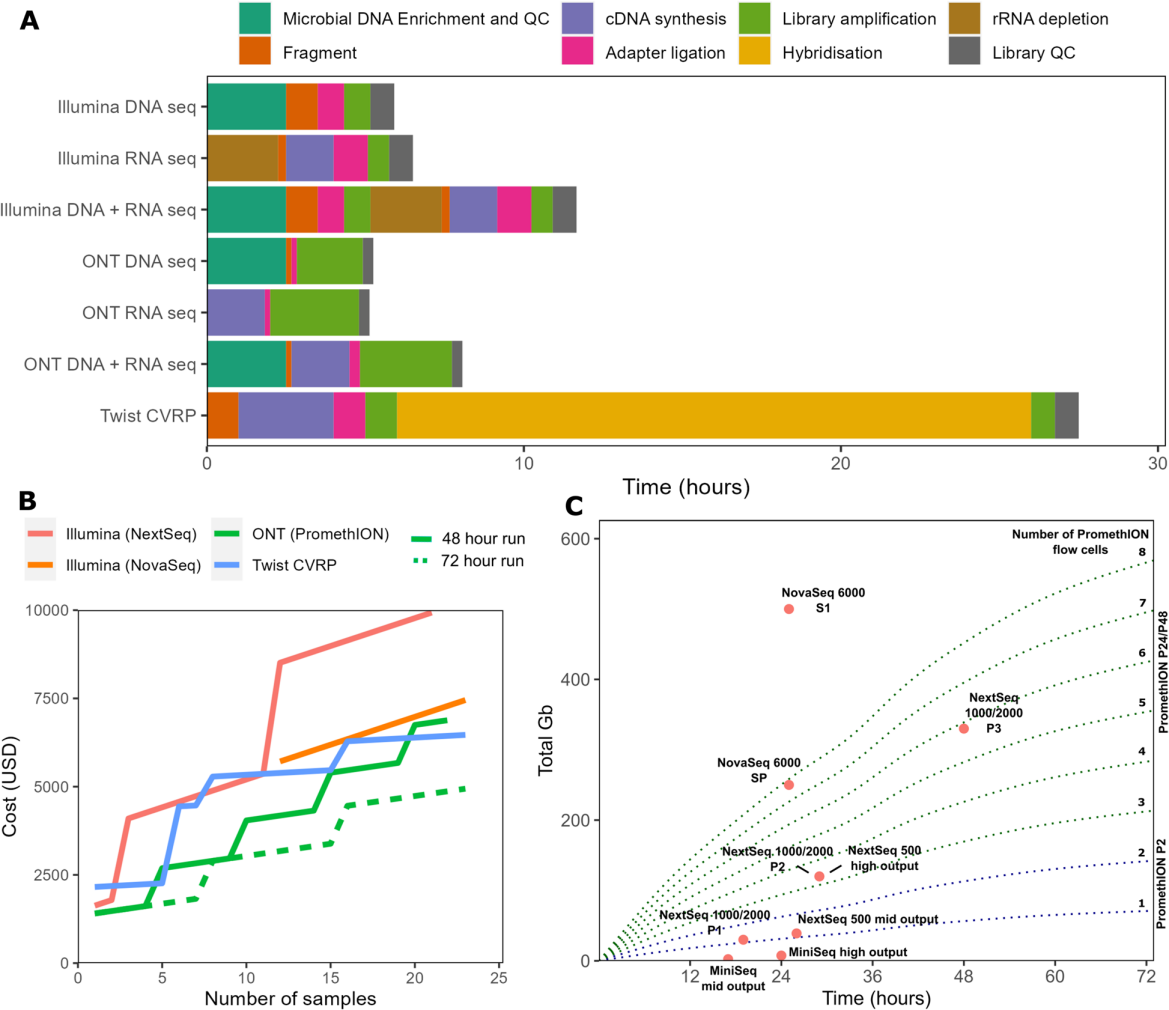
Turnaround times and output data volumes. **A** Time taken for library preparation for the different protocols tested. The Twist panel uses a combined DNA and RNA-Seq protocol. The DNA + RNA bars for the untargeted sequencing indicate the time taken if both protocols are performed by a single operator. **B** Total cost (including library preparation) to sequence number of samples indicated plus single negative control, to a depth of 5 GB. ONT costs are shown with 48- and 72-h maximum run times per flow cell. **C** Volume of data output by time for a range of Illumina sequencing kits and ONT sequencing with PromethION flow cells. The Illumina kits produce a set amount of data after the sequencing run is complete—this is shown by pink dots. In ONT sequencing, data is output continuously and the run can be stopped at any time, until the flow cell becomes degraded. PromethION data (green/blue dotted lines) shows the average of our RNA and DNA-Seq runs, passed reads only. Data outputs for Illumina were obtained from the product specification data as of April 2024

Costs and turnaround times from sample to results affect the adoption of metagenomics for routine diagnostics. ONT provides the quickest library preparation method, at just over 5 h for both DNA and RNA protocols (Fig. 5A). Targeted sequencing with the Twist CVRP requires overnight hybridization and is the slowest protocol (Fig. 5A). The Twist CVRP protocol was the cheapest based on 23 samples (+ negative control) and a sequencing depth of 5 Gb, while the untargeted Illumina sequencing was the most expensive (Fig. 5B, Table S7).

However, directly comparing the costs of each protocol to obtain at least 5 Gb of sequence data does not account for differences in their sensitivity. Since the Twist CVRP approach is at least 10–100 × more sensitive than untargeted Illumina and 100–1000 × more than untargeted ONT (Fig. 2), increases of orders of magnitude in sequencing depth would be required to bring the sensitivity of the untargeted protocols in line with that of the Twist CVRP. Even up to twice as much sequence data (10.6 and 11.3 Gb at 60 gc/ml) did not increase the sensitivity of untargeted Illumina and ONT respectively to anything near to the Twist CVRP. For untargeted Illumina, greater sequencing depth also amplifies the detection of contaminants, making interpretation more difficult. Achieving increased sensitivity using ONT sequencing would require long sequencing runs and a reduction in the number of samples sequenced per flow cell, significantly increasing costs and turnaround times. This means that targeted metagenomics methods such as Twist CVRP are by far the quickest and most cost-effective of the protocols for detection of low viral loads (60–600 gc/ml). Similarly, since untargeted Illumina is more sensitive than this untargeted ONT protocol, it will be quicker and cheaper to reach the sequencing depths required to detect intermediate viral loads (600–6000 gc/ml) using Illumina.

Sequencing costs and turnaround times will also be influenced by the number of samples. For fewer than six samples, including controls, ONT is the cheapest and fastest alternative where microbial load is likely to be high and genomic sequences are achievable with lower sequencing depth, for example 5 Gb of data, per sample (Fig. 5B, C). ONT also provides access to the sequencing data in real time, allowing preliminary analysis of the results before the run is completed, which can be advantageous for samples with high viral loads. However, if more samples are processed in parallel or a higher sequencing depth is required to improve sensitivity to a level comparable with untargeted Illumina, longer sequencing runs will be needed (Fig. 5B, C). When the total volume of data required per run is higher than around 30 Gb, it may be faster to use Illumina sequencing (Fig. 5C). However, it remains cheaper to use ONT with 23 sample runs (24 including negative control, 120 Gb) (Fig. 5B). Because of the Twist CVRP’s improved sensitivity, lower sequencing depths are required per sample, allowing the use of smaller Illumina sequencers and cheaper kits with shorter sequencing times (Fig. 5C). However, for fewer samples the Twist CVRP method may be much more expensive, since the kit optimal cost per sample is based on the pooling of 7 (8 including negative control) samples per hybridization (Fig. 5B, Table S6).

## Discussion

The use of metagenomics and allied targeted methods for routine diagnostics and clinical management are now priorities for laboratories in many countries. At least two commercial solutions are already available, in both cases using Illumina platforms for untargeted sequencing of cell-free DNA in blood, to identify causes of sepsis [104, 105]. However, these approaches may not be suitable for the detection of cell-associated pathogens, notably viruses, and data on limits of detection for viruses is absent. Untargeted Illumina sequencing is also in routine use in a handful of labs for the management of patients with fever of unknown origin, encephalitis, meningitis, and sepsis [10, 11, 106]. Most recently, routine diagnostic ONT metagenomic sequencing of respiratory samples has been proposed for improved management of critically ill patients with pneumonia [12, 13]. In each case, the metagenomic set-ups are multi-step workflows where each stage, from sample collection to computational data analysis, significantly affects the outcome of the test [26, 28, 107] (Fig. 1A). Sensitivity, specificity, reproducibility, turnaround time and cost are critical considerations before implementation in a clinical laboratory. However, with limited standardization across workflows, few head-to-head comparisons and significant, if underreported, drawbacks to most of the existing pipelines, choosing and implementing a metagenomics workflow remains complicated and uncertain for most.

In this study, we have focused on detection of viruses, which are particularly important causes of morbidity and mortality in immunocompromised patients [41–43]. Sensitive detection of viral infections is also necessary where metagenomics is being considered for screening of biological therapies such as blood and organ donations [44] and for detection of pathogens of high consequence, for example in returning travelers [45, 46]. Detection of viruses also has implications for antimicrobial stewardship and with increasing antiviral agents available, the appropriate stratification of patient management. Several studies have previously compared Illumina and ONT-based metagenomics of bacterial and fungal mock communities [29, 30, 32], simulated bacterial datasets [32, 33, 38] and clinical samples [18, 28, 108, 109]. Some work on viral detection from clinical samples [27, 110–114] or mock communities resembling environmental samples [34] has also been reported. While the sensitivity of both platforms, where compared, has been found to be similar for bacterial detection [27, 29, 30, 38, 110, 115], few have compared detection of RNA viruses. Recently, a multicenter study benchmarking 11 clinical metagenomic workflows using a panel of simulated low biomass samples, including CSF and nasopharyngeal swabs, tested different viral loads and showed that only a minority of protocols, including a Twist CVRP approach, were able to detect viruses at CT values of over 35 [47]. However, to our knowledge, no studies have systematically tested different viral loads, established limits of detection or specificity for viral detection and evaluated the quality of host transcriptomics information in samples with high human background.

Here we show that untargeted Illumina and ONT metagenomics, and targeted Illumina sequencing with the Twist CVRP, detect high viral loads (60,000 gc/ml) with good sensitivity and reproducibility. Metagenomic assembled genomes were generally low in quality, demonstrating the challenge of effectively de novo assembling low-level viral genomes amongst a background of human and contaminant sequences (Fig. S3). Untargeted Illumina sequencing appears better able than ONT to detect viruses at lower genome copy numbers, with the former finding all six viruses at 6000 and five at 600 gc/ml, while the latter detected only four and two of the six viruses respectively, with only untargeted Illumina finding a single virus at 60 gc/ml (Fig. 2). Notably ONT detected only two of the four RNA viruses at 6000 gc/ml, one of the four at 600 gc/ml and none at 60 gc/ml. This may be because depletion of ribosomal RNA (rRNA) before performing Rapid-SMART-9N [116], which is known to improve Illumina detection of RNA viruses, resulted in levels of RNA input that are too low for adequate ONT library preparation. In order to overcome this, adapting the current workflow to include cDNA synthesis kits compatible with ultra-low input RNA, such as those used for single-cell RNA-seq experiments, should be considered, which could improve the sensitivity of ONT, particularly for single-stranded (ss) RNA viruses. Combining ONT with differential lysis methods, which remove host and non-encapsulated nucleic acids, can improve sensitivity [117, 118] detection of bacteria and fungi, but this step may reduce sensitivity for certain microbes and reduce the ability to detect cell-free DNA and RNA, including viral nucleic acid [54, 55]. Furthermore, with increasing moves to combine host gene expression with microbial detection to improve infection-diagnosis rates [27, 50–53], methods such as differential lysis, which deplete human nucleic acid may be less attractive.

More sensitive than either untargeted Illumina or ONT, viral enrichment using the commercially available Twist CVRP panel was able to detect all six viruses down to levels of 60 gc/ml, a finding in keeping with reports for other commercial capture protocols [19]. However, the Twist CVRP only includes viral probes and may require the addition and evaluation of probes targeting other pathogens and AMR genes to be useful for routine diagnostic use, since a virus-only panel does not allow syndromic diagnosis of infection. Having a defined panel may also limit the ability to detect novel pathogens. The probes can detect organisms with up to 20% difference to the reference with over 50% coverage [119], but cannot detect more divergent infectious agents, as exemplified by the failure of the Twist CVRP to detect the internal control *E. coli* phages MS2 in certain samples where it was detected in all untargeted Illumina runs (Table S3). However, as demonstrated by the host transcriptomic analysis of the Twist CVRP data, non-targeted material is retained by this protocol. This means that it may be possible to detect non-targeted microbial species, including bacteria, fungi and highly divergent viruses, if their abundance is high enough in relation to the depth of sequencing used. Finally, capture probe-based methods are currently designed only for use with Illumina sequencing. Previously reported attempts to add an enrichment step to improve the sensitivity of ONT sequencing require first generating an Illumina sequencing library before converting this for ONT sequencing through additional library preparation steps [22], making this approach costly and time-consuming.

The propensity for deep sequencing metagenomic methods to detect contaminant species presents a particular challenge when such methods are considered for routine diagnostic use. The numbers of falsely detected species were lowest for ONT sequencing and greatest for untargeted Illumina sequencing and the Twist CVRP (Fig. 3B). The higher precision of ONT is most likely due to longer reads making it easier for taxonomic classifiers to unambiguously assign reads to species. Large numbers of false positive species were identified for untargeted and targeted (Twist CVRP) Illumina sequencing. This was particularly pronounced for commonly used classifiers for bacterial data such Kraken2, whose kmer-based approach can result in inaccurate assignment of short reads due to cross-mapping (Fig. S6) [39]. Although the results of Kraken2 can be improved by post-processing with Bracken, this approach has a lower sensitivity than classifiers such as metaMix and CZ ID.

By contrast, the use of probabilistic methods that inherently control false positives, such as metaMix, reduced the numbers of false positive species to levels similar to those seen for ONT (Fig. 3C). By applying thresholds based on a combination of reads per million ratio, which compares species detected in samples and corresponding negative controls, and proportion of microbial reads, we demonstrate that false positive rates can be reduced for all classifiers, thus standardizing outputs from different sequencing methods and classifiers. Our approach differs from those previously applied, where only one of these measures or raw read counts alone was used. Our method highlights the importance of sequencing negative controls, which can help remove contaminants, particularly those present in the reagents. Using this approach, our results suggest capture panels, such as the Twist CVRP, provide the best sensitivity and specificity for routine detection of viruses, albeit with the caveats discussed above. Importantly, we show that the use of suitable taxonomic classifiers or appropriate thresholds based on comparison with the negative control and the proportion of the total reads assigned to that species overcomes the low specificity that has previously been reported for the Twist CVRP when used with its recommended One Codex platform (Fig. 3) [21].

Our study demonstrates the need for better control materials across platforms. Negative controls should resemble the true samples as much as possible, particularly in terms of human nucleic acid content, and blank extraction controls may be insufficient. Further work is needed to identify suitable internal controls, which should be viruses that cannot easily be mistaken for clinically relevant species. DNA internal controls should be reliably detected with ONT and correctly classified bioinformatically. For targeted approaches, the controls chosen should be targeted by the panel to allow uniform detection in all the samples tested, facilitating result interpretation.

Host transcriptomic data obtained from untargeted Illumina sequencing has been shown to help distinguish between bacterial and viral infection and infectious and non-infectious causes of disease by identifying the host immune pathways that are upregulated which could help to confirm negative or inconclusive results from pathogen identification [50, 51]. The Twist CVRP and ONT show relatively good agreement with the untargeted Illumina protocol’s estimates of human gene expression, although ONT fails to detect some low-abundance genes (Fig. 4). It is therefore likely that useful transcriptomic information may be obtained from any of the protocols, providing a method that preserves human RNA is selected. The analysis remains possible with the Twist CVRP because non-targeted DNA/RNA sequences are retained in an unbiased way, even though the targeted viral sequences are enriched. Since commercial total brain RNA with no particular infection was used to generate the mock samples for this analysis, it was only appropriate to compare quantification of transcripts genome-wide. Further work is now required to validate the performance of ONT and targeted Illumina sequencing in clinical samples, particularly their ability to detect the expression of immune pathways.

Both turnaround times and cost are critical parameters when considering the introduction of new diagnostic methods. Targeted sequencing with the Twist CVRP was the only viable method we tested for detection of low viral loads (60 gc/ml), since increasing the depth of untargeted sequencing by the orders of magnitude required to match the sensitivity of the Twist CVRP is too expensive and time-consuming to be practical. If an untargeted approach is required, perhaps to test for bacteria and other microbes as well as viruses in a single test, ONT can provide rapid results in cases where sample numbers are low and viral loads are high. In most other circumstances, Illumina is currently the quicker and cheaper way to produce the volumes of data required, particularly as higher volumes of ONT data are required to give the same level of sensitivity. Illumina sequencing may also allow more reliable quantification of human gene expression, making it easier to rule out infection when no pathogens are found.

Our study has several limitations. Since we used commercially available purified nucleic acid standards, we do not compare extraction protocols, which have been shown to have a large impact on the results of metagenomics [120, 121]. Different approaches have been used to reduce host content in samples in efforts to improve sensitivity. Pre-purification methods like filtration and centrifugation can efficiently remove human cells. However, they can significantly reduce sensitivity for cell-associated viruses [122–124]. Alternatively, differential lysis-based methods, which rely on selectively lysing human cells either using mechanical methods such as bead-beating [49] or with saponin [125], have been used to deplete human DNA and RNA prior to ONT sequencing. However, these approaches can lead to biases in organisms detected and reduce detection of cell-free DNA, which may arise from organisms killed by the immune system or antibiotics [54]. Additionally, any protocol that removes host material during or before the lysis steps, may lead to reduced sensitivity for integrated and intracellular viruses [54, 55]. These approaches could also be used prior to Illumina sequencing, although they will prevent host transcriptomic analysis.

Furthermore, we focused only on viruses, while the key advantage of metagenomics is its ability to detect all organisms. Although several studies have shown similar sensitivity to bacteria for Illumina and ONT sequencing on mock communities [29, 30], further work is needed to compare commonly used methods such as Illumina sequencing of cell-free DNA and ONT sequencing with differential lysis for detection of bacteria and eukaryotic microbes. We focus only on sterile site samples with high host content such as tissue and whole blood. We expect that different laboratory and bioinformatics methods will also be appropriate for non-sterile sites such as respiratory samples and for samples with low biomass such as plasma and CSF.

## Conclusions

Different metagenomics platforms perform best in terms of sensitivity, specificity, and turnaround times, with no single test currently being optimal in all clinical contexts. Where sensitivity for viral detection is less of a consideration, as might be the case for respiratory samples from severely ill patients with pneumonia, ONT is faster and cheaper. Target capture approaches with Illumina may be preferred for samples with low microbial diversity, where high sensitivity for both DNA and RNA viruses is required to reliably confirm or exclude infection, for example in immunosuppressed patients with fever or encephalitis, blood products and where high consequence pathogens are suspected. Development of rapid, commercially available targeted methods for a wide range of pathogens for both long- and short-read platforms, using methods that preserve the host transcriptome and also allow rapid untargeted metagenomics where required for pathogen discovery, will bring us closer to a diagnostic test that can detect any pathogen in an actionable timeframe and that could revolutionize clinical microbiology.

## Supplementary Information

Additional file 1: Supplementary Figures S1-S6.

Additional file 2: Supplementary Information: More detailed description of derivation of thresholds for calling positive species.

Additional file 3: Table S1: Viral mock community quantification.

Additional file 4: Table S2: Sequencing summary.

Additional file 5: Table S3: Internal controls.

Additional file 6: Table S4: Raw results.

Additional file 7: Table S5: Genome assembly.

Additional file 8: Table S6: False positive viruses obtained after application of thresholds.

Additional file 9: Table S7: Cost comparison.

Additional file 10: Table S8: False positive viruses obtained after application of thresholds.

## Data Availability

The raw data produced in this study is available in the European Nucleotide Archive (ENA) with accession PRJEB74559 (https://www.ebi.ac.uk/ena/browser/view/PRJEB74559) [126]. Table S8 shows the mapping for ENA IDs to our samples. All scripts used for analysis are available at https://github.com/sarah-buddle/viral-metagenomics-comparison. The method for applying thresholds to the raw outputs of taxonomic classifiers is available as an R package at https://github.com/sarah-buddle/metathresholds.

## Abbreviations

ONT: Oxford Nanopore Technologies
CVRP: Comprehensive Viral Research Panel
