# Supplementary material for: Evaluating metagenomics and targeted approaches for diagnosis and surveillance of viruses
Source: Genome Med. 2024 Sep 9;16:111. doi: 10.1186/s13073-024-01380-x (PMC11382446; doi:10.1186/s13073-024-01380-x)
Supplement: Supplementary file 1 — Additional file 1: Supplementary Figures S1-S6. [file 13073_2024_1380_MOESM1_ESM.pdf]

Supplementary Figures

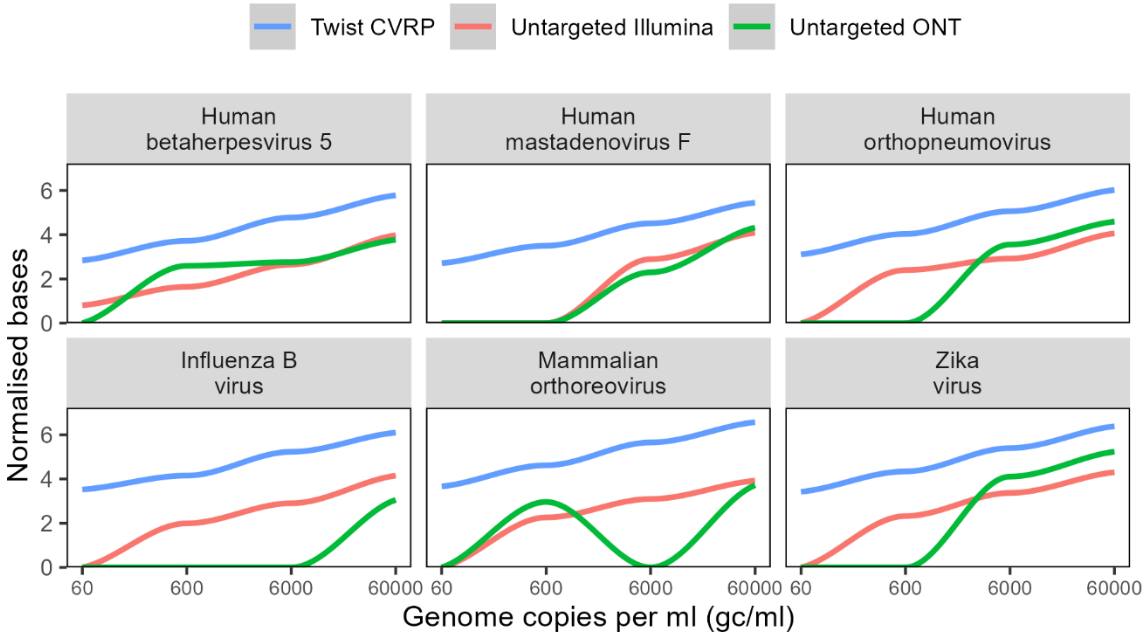

Figure S1: Identification of species in mock community normalized by genome length

Normalised bases aligning to the genome of each species in the mock community, calculated as log10(base pairs * 10^4 / genome length).

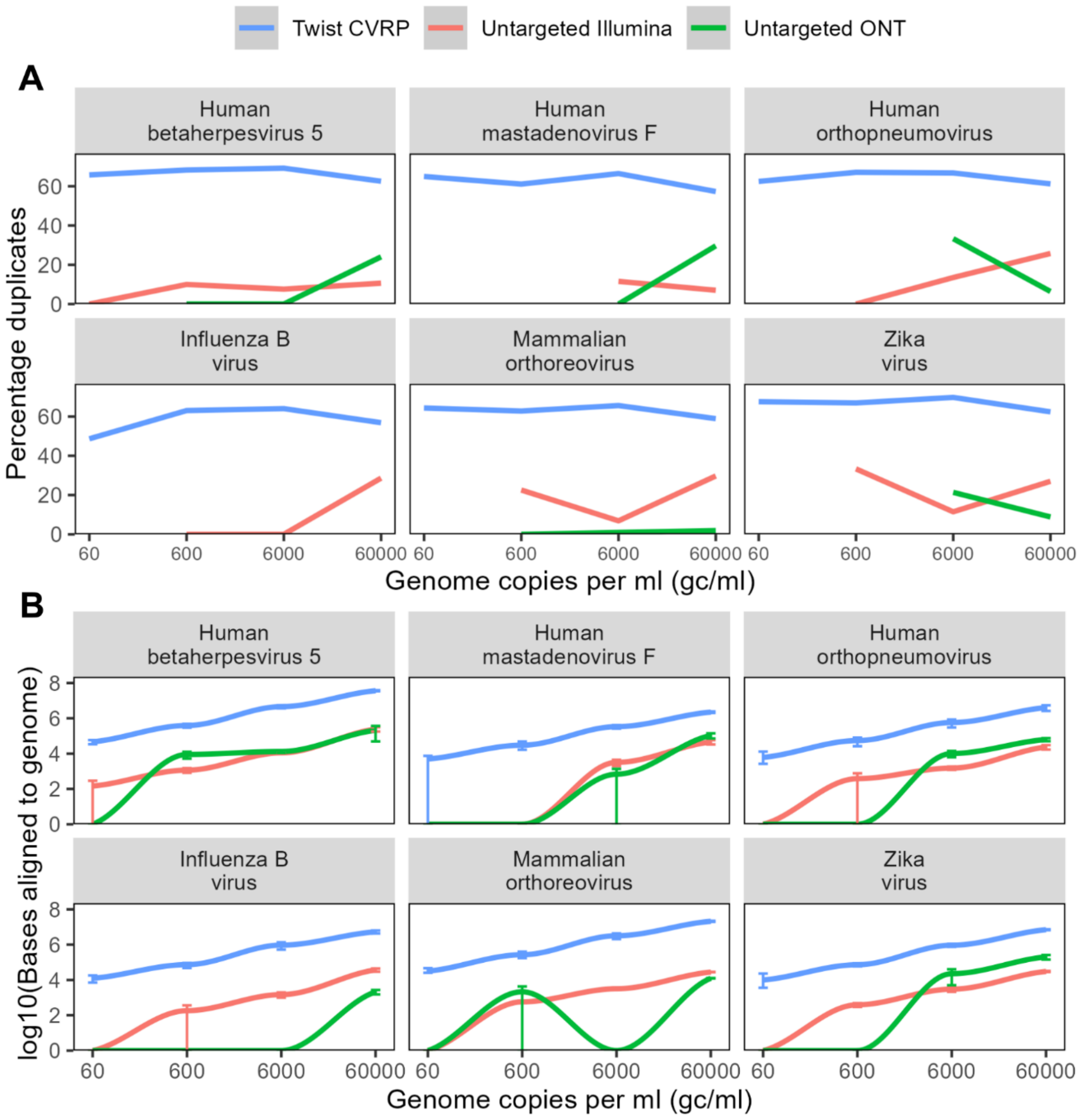

Figure S2: PCR duplicates

A Percentage of the total reads aligning to the genome of each species that were marked as duplicates by samtools markdup. **B** Figure 2B without PCR duplicates removed.

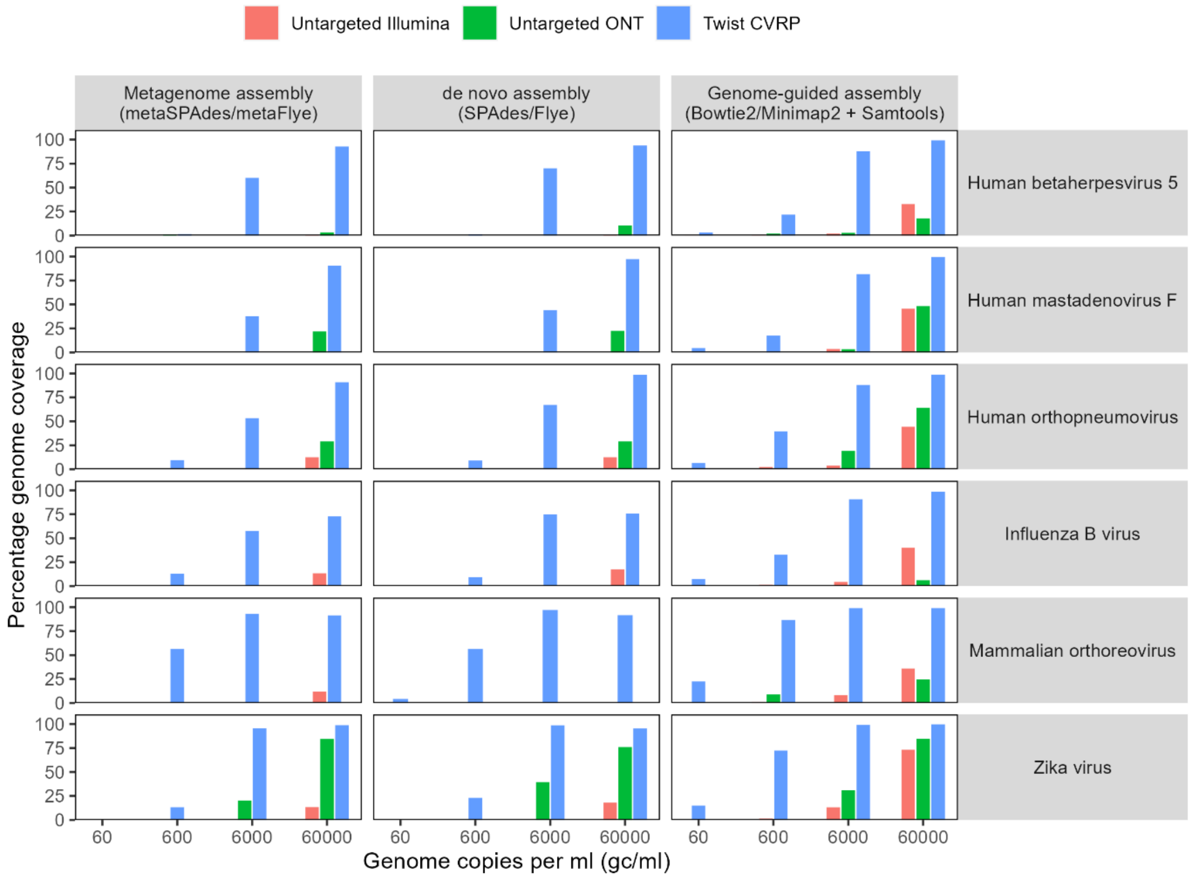

Figure S3: Genome assembly of mock community species

Genome coverage of species in mock community by aligning contigs from metagenomic assembly to viral genomes (metagenome *de novo* assembly), assembling reads that aligned to viral genomes (*de novo* assembly) and generation of consensus genomes from alignments (genome-guided assembly). Values shown are mean of at least two technical replicates, where contigs were found in more than one replicate.

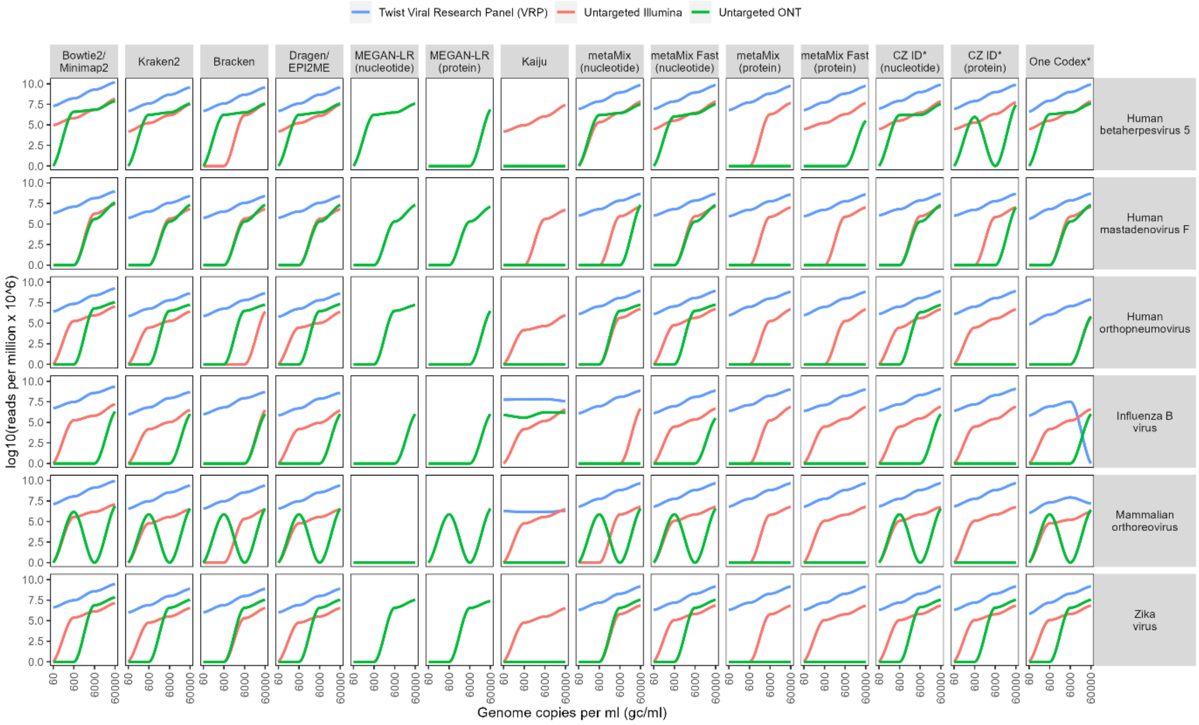

Figure S4: Reads classified to mock community by different taxonomic classifiers

Number of reads assigned to each species in the mock community by classifiers. Each point shows the mean of at least two technical replicates.

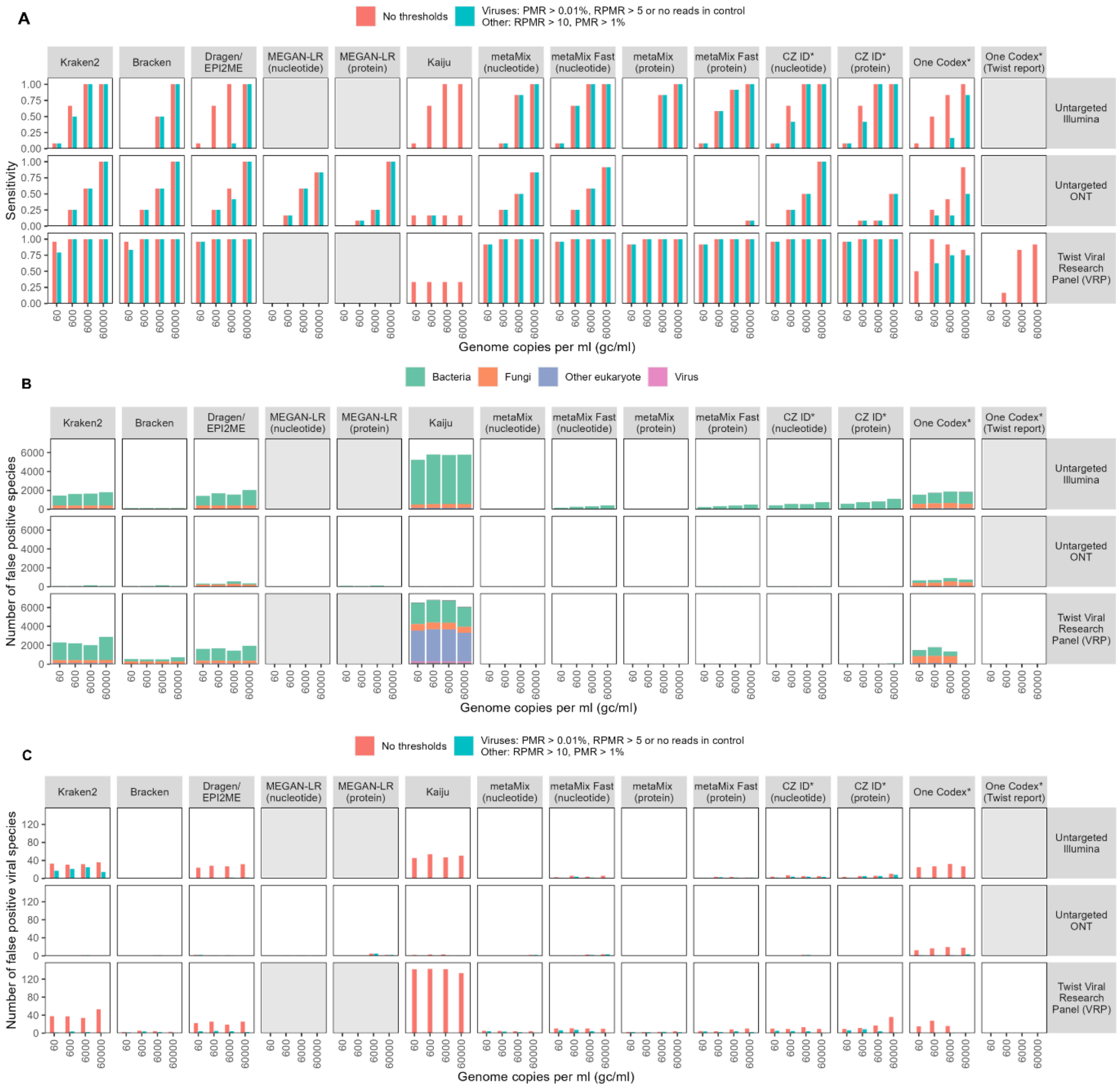

Figure S5: Sensitivity and false positive species identified with nucleotide and protein-based classifiers

A Sensitivity to the species in the mock community before and after the application of thresholds, for eight different taxonomic classifiers, including their protein modes where relevant, by untargeted Illumina and ONT sequencing and capture probe enrichment with the Twist Bioscience Comprehensive Viral Research Panel followed by Illumina sequencing. MEGAN-LR and the One Codex Twist report are only designed for ONT and Twist sequencing respectively so were only run for these platforms. **B,C** Number of false positive species, defined as a species that is classified as positive but not present in the mock community **B** False positive species from the raw output of the taxonomic classifiers with no thresholds applied. **C** Comparison of the numbers of viral positive species identified before and after the application of thresholds. Genome copy numbers refer to an average across the viral species – see **Supplementary Table 6**. Each bar shows the mean of at least two technical replicates.

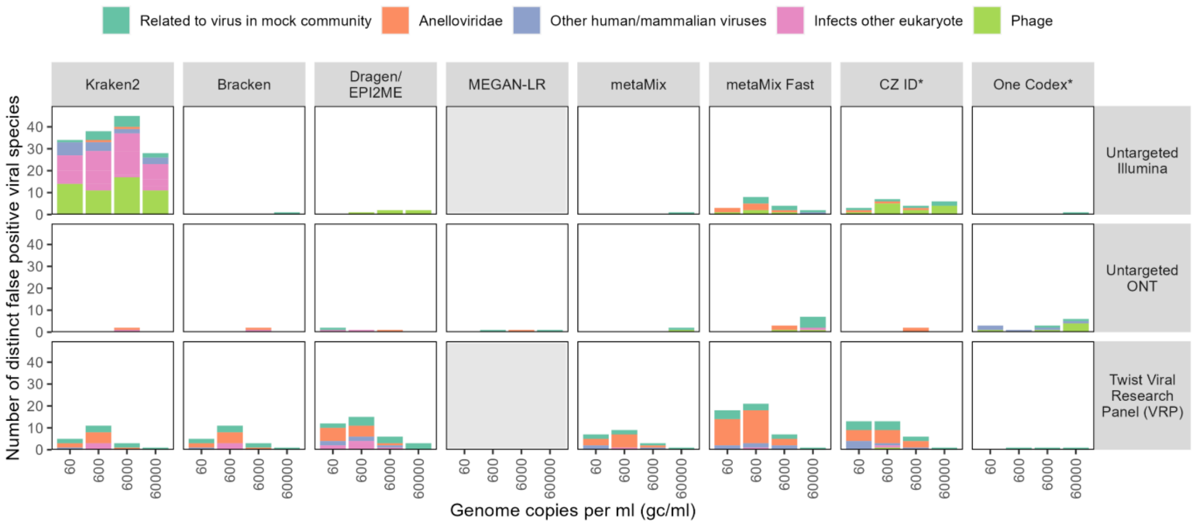

Figure S6: False positive viruses

Number of distinct false positive viral species, defined as a species that is classified as positive but not present in the mock community or positive controls, after application of thresholds described in **Figure 3**. Shows the number of distinct species across the 2-4 technical replicates, so totals may be slightly higher than in **Figure 3C**, which shows an average across technical replicates.
